# Treatment-resistant depression with poor response to deep brain stimulation improves with psychotherapy: case series

**DOI:** 10.1186/s12991-025-00588-4

**Published:** 2025-09-30

**Authors:** Shuping Fang, Yingyi Kang, Jun Zhang

**Affiliations:** 1 Mental Health Center of West China Hospital, Sichuan University, No. 37, Guo Xue Alley, Chengdu, Sichuan Province 610041 China; 2 The Fourth People’s Hospital of Ya’an, Ya’an, China

**Keywords:** Treatment-resistant depression, Deep brain stimulation, Cognitive behavioral therapy, Psychotherapy

## Abstract

**Background:**

Deep brain stimulation (DBS) is regarded as an efficacious treatment for treatment-resistant depression (TRD), exhibiting a response rate of approximately 60%. However, certain patients exhibit limited responsiveness to DBS, necessitating further exploration of alternative interventions. In this paper, we present two cases of TRD patients who exhibited poor response to DBS surgery but showed significant improvement after receiving psychotherapy.

**Case presentation:**

In case 1, a 20-year-old female patient exhibited a slight initial positive response after DBS surgery; however, her symptoms continued to deteriorate progressively. Following systematic cognitive behavioral therapy (CBT), she demonstrated remarkable improvement in depressive symptoms. In case 2, a 36-year-old male patient experienced short-term symptomatic improvement post-DBS surgery but relapsed due to treatment interruption caused by financial constraints. After CBT intervention, the symptoms of his depression exhibited significant improvement.

**Conclusions:**

No previous studies have reported on the effects of CBT in postoperative depressive symptoms following DBS. The combination of DBS surgery and postoperative psychological therapy may enhance the therapeutic outcomes of DBS. This study emphasizes the significance of incorporating psychotherapy into the management after DBS surgery and calls for future research to further investigate the potential and mechanisms underlying this comprehensive treatment strategy.

## Background

Treatment-resistant depression (TRD) is commonly defined as the lack of response to two or more full-dose and full-course antidepressant regimens while adhering to treatment, accounting for at least 30% of depression [1]. This clinical scenario has prompted the exploration of deep brain stimulation (DBS) as a potential therapeutic approach [2]. DBS is considered an effective intervention for TRD, demonstrating a response rate of 60% [2]. However, the current surgical technology for DBS is still in its developmental stage, and some patients exhibit poor responsiveness to this treatment modality, necessitating further investigation into potential solutions [3]. In this paper, we present two cases of TRD patients who exhibited limited improvement in depressive symptoms following DBS surgery but demonstrated significant amelioration after receiving postoperative psychotherapy.

### Case presentation

### Case 1

A 20-year-old female from China has been suffering from depression for over three years. The patient, accompanied by her mother, had been hospitalized more than ten times due to persistent depressive symptoms that proved refractory to multiple pharmacological treatments. She had received adequate trials of several antidepressants—fluoxetine, venlafaxine, duloxetine, maprotiline, and bupropion—with minimal clinical benefit. As the illness progressed, episodic mood fluctuations and poor treatment response prompted a diagnostic reassessment, ultimately leading to a diagnosis of bipolar disorder. Despite long-term lithium therapy, the patient continued to experience prominent depressive symptoms, along with persistent suicidal ideation and self-harm behaviors. Subsequent augmentation strategies, including valproate magnesium, lamotrigine, quetiapine, olanzapine, and clozapine, were ineffective. She also underwent 12 sessions of modified electroconvulsive therapy (MECT), with only limited therapeutic response. In July 2023, she underwent MR-guided focused ultrasound (MRgFUS) treatment at a hospital in Shanghai, but the results were limited. In October 2023, she received bed nucleus of the stria terminalis (BNST)-nucleus accumbens (NAc) DBS at the same hospital (stimulation parameters: voltage 3 V, pulse width 210 µs, frequency 170 Hz). Despite an initial transient positive response to DBS, her condition continued to progressively deteriorate.

Upon admission to our hospital in January 2024, the patient was prescribed a medication regimen consisting of sodium valproate 500 mg/day, clozapine 150 mg/day, and fluoxetine 60 mg/day. During her hospitalization period, we implemented a systematic cognitive behavioral therapy (CBT) approach. CBT sessions typically began around 9:00 AM, lasted 45 min, and were conducted four times per week. A pivotal aspect of her treatment involved collaboratively establishing a daily self-assessment routine performed no less than twice per day to evaluate the intensity of suicidal impulses on a scale from 0 to 10 points and the level of emotional self-management on a scale from 0 to 10 points. This strategic intervention facilitated enhanced recognition and monitoring of emotional fluctuations and suicide risk, thereby promoting effective communication among the patient, her mother, and healthcare professionals.

After six psychotherapy sessions, the patient demonstrated improved recognition of mood fluctuations, though suicide and self-harm risks remained. To further mitigate these risks and promote social functioning, we established a timeline for the absence of her mother and implemented “safe solitary training”. We provided guidance in finding a balance between self-assurance in solitude and apprehension of losing control, engaging in daily activities to foster emotional regulation and independent living. Throughout a five-month follow-up period, the patient gradually extended the duration of her alone time. The behavioral training not only ensured her safety but also bolstered her self-confidence, alleviated feelings of guilt, and enhanced her emotional and behavioral responses across various scenarios, thereby augmenting her resilience towards setbacks.

### Case 2

A 36-year-old male from China has been battling depression for 11 years, with approximately 20 hospitalizations during this period. He had received adequate courses of various psychiatric medications, including venlafaxine, paroxetine, maprotiline, and alprazolam, alongside modified ECT. Despite these treatments, his condition continued to deteriorate. In November 2022, he underwent DBS surgery at our hospital’s neurosurgery department. Although the patient could not recall the specific parameters used during the procedure, he reported an immediate improvement in mood and resolution of insomnia post-surgery. However, eight months later, due to financial constraints, he discontinued DBS treatment which led to a rapid relapse of depressive symptoms significantly impairing his daily functioning.

In November 2023, due to a relapse characterized by severe depressive symptoms and emergent suicidality, the patient was rehospitalized. At the time of admission, his medication regimen consisted of venlafaxine 225 mg/day, paroxetine hydrochloride 40 mg/day, and doxepin hydrochloride 25 mg/day. During his hospital stay, evidence-based health education was initiated to emphasize the effectiveness of psychotherapy and pharmacotherapy for TRD, even without DBS surgery. A systematic CBT approach was employed, with sessions typically starting in the morning, lasting 45 min, and conducted four times per week. Behavioral activation therapy was prioritized to address depression by rediscovering the patient’s interests and hobbies such as singing and playing table tennis.

**Fig. 1:**
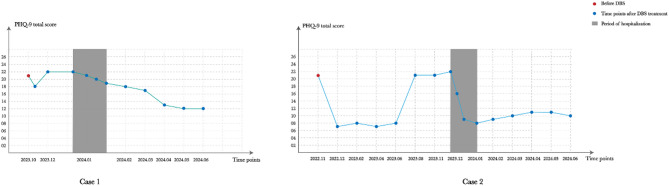
The PHQ-9 scores of 2 patients during the follow-up period after discharge

After one week of treatment, the patient gradually developed an interest in singing and playing table tennis. We identified perfectionistic tendencies in the patient, so we devised innovative behavioral tasks, such as intentionally singing off-key and with incorrect lyrics, as well as purposefully subjecting oneself to embarrassment in group settings, in order to enhance the pleasure derived from singing and challenge the core belief of perfectionism. These strategies facilitated emotional release for the patient. Through this comprehensive approach, distorted cognitions were rectified and the patient acquired skills for engaging in relaxed social interactions and effectively managing negative emotions. Following a five-month follow-up period, the patient reported a restoration of their pre-illness state, enabling them to actively participate in work with a positive outlook. The patients continued to receive regular follow-ups and individualized psychotherapy after discharge. The post-DBS PHQ-9 scores are depicted in Fig. 1. Additionally, Tables 1 and 2 separately summarize the PHQ-9 scores of Case 1 and Case 2, outlining their quantitative progression across all clinical phases.

Each data point represents the PHQ-9 total score assessed at a specific timepoint. Red dots indicate scores obtained prior to DBS implantation; blue dots represent follow-up assessments after DBS initiation. The gray shaded area denotes the period of hospitalization.

**Table 1:** PHQ-9 scores for case 1 across all clinical phases

Date	Timepoint Description	PHQ-9 Score
2023.10	Pre-DBS	21
2023.11 2023.12	Post-DBS Post-DBS	18 22
2024.01 2024.01 2024.01 2024.01	During hospitalization During hospitalization During hospitalization During hospitalization	22 21 20 19
2024.02 2024.03 2024.04 2024.05	Follow-up Follow-up Follow-up Follow-up	18 17 13 12
2024.06	Follow-up	12

Longitudinal PHQ-9 scores for Case 1, showing depression severity across preoperative, hospitalization, and follow-up phases relative to DBS surgery

**Table 2:** PHQ-9 scores for case 2 across all clinical phases

Date	Timepoint Description	PHQ-9 Score
2022.11	Pre-DBS	21
2022.12	Post-DBS	7
2023.02 2023.04 2023.06 2023.08	Post-DBS Post-DBS Post-DBS Post-DBS	8 7 8 21
2023.11 2023.12 2023.12 2023.12 2024.01 2024.02	Post-DBS During hospitalization During hospitalization During hospitalization During hospitalization Follow-up	21 22 16 9 8 9
2024.03 2024.04 2024.05 2024.06	Follow-up Follow-up Follow-up Follow-up	10 11 11 10

Longitudinal PHQ-9 scores for Case 2, capturing the trajectory of PHQ-9 scores before and after DBS, including hospitalization and follow-up assessments

## Discussion

DBS modulates pathological neural circuits and regulates information transmission by implanting electrodes in specific brain structures [3]. By manipulating the opening and closing of voltage-gated sodium channels on neurons, DBS utilizes electric fields to induce action potentials [4]. These action potentials further regulate neurotransmitter release in targeted pathways, triggering a cascade of cellular, molecular, and neuroplasticity changes that exert multi-level effects on the nervous system [4]. However, the exact mechanism through which DBS improves depressive symptoms in TRD remains to be fully elucidated. Existing studies have not yet identified the optimal stimulation target or parameters for DBS in TRD treatment nor determined which subgroups of TRD patients would benefit most from DBS therapy(3). Currently, DBS surgery is not recommended as a standard clinical intervention [5], with its implementation limited to clinical trials only [2].

Psychotherapy is increasingly regarded as an effective treatment for depression and may enhance the efficacy of concurrent therapies [6, 7]. Adding psychotherapy to usual care, including pharmacotherapy, has been shown to improve depressive symptoms and increase response and remission rates in patients with TRD [8]. However, there is limited evidence supporting the superiority of one psychotherapeutic modality over another [9]. Nonetheless, a large European cross-sectional observational study found that combining manual-driven psychotherapy (MDP) with medication is not associated with better treatment outcomes in patients with TRD [10].

CBT has been extensively researched as a psychotherapeutic approach for depression, including TRD [11]. Previous randomized controlled trials have shown that CBT, as an adjunct to standard care including antidepressants, is beneficial for TRD [12, 13] and cost-effective in the long term [14]. A meta-analysis further indicated that CBT offers superior efficacy in patients with TRD, with effects that persist over time [15]. Although the exact mechanisms of psychotherapy remain unclear, research has demonstrated that cognitive reconstruction intervention based on cognitive therapy in CBT can mitigate the inclination to attribute negative events solely to internal causes, while goal-setting intervention grounded in behavioral activation therapy can diminish sensitivity towards effort [16]. This behavioral intervention may stimulate a positive reinforcement cycle that ameliorates mood [16]. It should be noted that behavioral activation therapy (BAT), which incurs lower costs compared to CBT, could also serve as a standalone treatment option and potentially yield similar effects as CBT in treating adult depression [11].

Currently, psychotherapy is frequently overlooked in patients with TRD [7]. However, global investment in mental health remains insufficient, particularly in Asia [17]. It is noteworthy that CBT demonstrates superior efficacy in patients from non-Western countries compared to Western countries [18], and exhibits a particularly significant effect on Chinese patients [19]. Nevertheless, due to cultural values, the majority of Chinese individuals are hesitant to seek face-to-face psychotherapy [20]. Furthermore, the effectiveness of CBT is also influenced by factors such as treatment modality and the professional competence of psychological counselors [19]. Overcoming these challenges may prove difficult for future depression treatments. Therefore, future research should focus on elucidating the mechanisms underlying psychotherapy’s action and developing personalized treatment options tailored to different cultural backgrounds and diverse populations.

This study is limited by its small sample size, which restricts the generalizability of the findings. Additionally, the confounding effect of ongoing pharmacological treatment may have influenced the observed outcomes, as the impact of combined interventions was not fully isolated. Lastly, the lack of standardized measures beyond the PHQ-9 limits the scope of our assessment of depressive symptoms.

Given these limitations, future studies should include larger, more diverse samples to improve generalizability. Controlling for pharmacological treatments and using standardized tools to assess multiple mental health dimensions, such as anxiety, cognition, and quality of life, would provide a more comprehensive understanding. Longitudinal studies with better control for confounders would clarify causal relationships between interventions and outcomes.

## Conclusion

The future of TRD solutions is characterized by resistance and longevity [1]. Currently, there is limited research on the postoperative management of psychiatric diseases in relation to DBS applications [21]. It should be noted that preoperative and postoperative management may hold equal importance as the DBS intervention itself [21]. Furthermore, DBS, being an invasive therapy involving active stimulation, might be perceived by patients as a “last resort” for rehabilitation [3]. However, some patients still exhibit poor response to DBS. Considering the distinct and complementary mechanisms of psychotherapy and DBS, we propose considering psychotherapy in the postoperative management of patients with inadequate response to enhance antidepressant treatment efficacy.

Notably, no previous study has investigated the impact of psychotherapies like CBT on depressive symptoms following DBS. Therefore, our case highlights the significant role of psychotherapy in alleviating depressive symptoms and underscores its promising potential in treating TRD.

## Data Availability

No datasets were generated or analysed during the current study.

## References

[1] McIntyre RS, Alsuwaidan M, Baune BT, Berk M, Demyttenaere K, Goldberg JF, et al. Treatment-resistant depression: definition, prevalence, detection, management, and investigational interventions. World Psychiatry. 2023;22(3):394–412. 37713549 10.1002/wps.21120 PMC10503923

[2] Figee M, Riva-Posse P, Choi KS, Bederson L, Mayberg HS, Kopell BH. Deep brain stimulation for depression. Neurotherapeutics. 2022;19(4):1229–45. 35817944 10.1007/s13311-022-01270-3 PMC9587188

[3] Hitti FL, Yang AI, Cristancho MA, Baltuch GH. Deep brain stimulation is effective for Treatment-Resistant depression: A Meta-Analysis and Meta-Regression. J Clin Med. 2020;9(9). 10.3390/jcm9092796 PMC7564277 32872572

[4] McIntyre CC, Anderson RW. Deep brain stimulation mechanisms: the control of network activity via neurochemistry modulation. J Neurochem. 2016;139(Suppl 1):338–45. 27273305 10.1111/jnc.13649 PMC5358920

[5] Wu Y, Mo J, Sui L, Zhang J, Hu W, Zhang C, et al. Deep brain stimulation in Treatment-Resistant depression: A systematic review and Meta-Analysis on efficacy and safety. Front Neurosci. 2021;15:655412. 33867929 10.3389/fnins.2021.655412 PMC8047101

[6] Guidi J, Fava GA. The clinical inadequacy of the concept of treatment-resistant depression: innovative strategies in assessment and psychotherapeutic management. Clin Psychol Rev. 2025;120:102616. 40580814 10.1016/j.cpr.2025.102616

[7] Markowitz JC, Wright JH, Peeters F, Thase ME, Kocsis JH, Sudak DM. The neglected role of psychotherapy for Treatment-Resistant depression. Am J Psychiatry. 2022;179(2):90–3. 35105164 10.1176/appi.ajp.2021.21050535

[8] Ijaz S, Davies P, Williams CJ, Kessler D, Lewis G, Wiles N. Psychological therapies for treatment-resistant depression in adults. Cochrane Database Syst Rev. 2018;5(5):Cd010558. 29761488 10.1002/14651858.CD010558.pub2 PMC6494651

[9] Rogan T, Wilkinson ST. The role of psychotherapy in the management of Treatment-Resistant depression. Psychiatr Clin North Am. 2023;46(2):349–58. 37149349 10.1016/j.psc.2023.02.006

[10] Bartova L, Fugger G, Dold M, Swoboda MMM, Zohar J, Mendlewicz J, et al. Combining psychopharmacotherapy and psychotherapy is not associated with better treatment outcome in major depressive disorder - evidence from the European group for the study of resistant depression. J Psychiatr Res. 2021;141:167–75. 34216945 10.1016/j.jpsychires.2021.06.028

[11] Ciharova M, Furukawa TA, Efthimiou O, Karyotaki E, Miguel C, Noma H, et al. Cognitive restructuring, behavioral activation and cognitive-behavioral therapy in the treatment of adult depression: A network meta-analysis. J Consult Clin Psychol. 2021;89(6):563–74. 34264703 10.1037/ccp0000654

[12] Wiles N, Thomas L, Abel A, Ridgway N, Turner N, Campbell J, et al. Cognitive behavioural therapy as an adjunct to pharmacotherapy for primary care based patients with treatment resistant depression: results of the CoBalT randomised controlled trial. Lancet. 2013;381(9864):375–84. 23219570 10.1016/S0140-6736(12)61552-9

[13] Nakagawa A, Mitsuda D, Sado M, Abe T, Fujisawa D, Kikuchi T, et al. Effectiveness of supplementary Cognitive-Behavioral therapy for Pharmacotherapy-Resistant depression: A randomized controlled trial. J Clin Psychiatry. 2017;78(8):1126–35. 28252882 10.4088/JCP.15m10511

[14] Wiles NJ, Thomas L, Turner N, Garfield K, Kounali D, Campbell J, et al. Long-term effectiveness and cost-effectiveness of cognitive behavioural therapy as an adjunct to pharmacotherapy for treatment-resistant depression in primary care: follow-up of the CoBalT randomised controlled trial. Lancet Psychiatry. 2016;3(2):137–44. 26777773 10.1016/S2215-0366(15)00495-2

[15] Li J-M, Zhang Y, Su W-J, Liu L-L, Gong H, Peng W, Jiang C-L. Cognitive behavioral therapy for treatment-resistant depression: A systematic review and meta-analysis. Psychiatry Res. 2018;268:243–50. 30071387 10.1016/j.psychres.2018.07.020

[16] Norbury A, Hauser TU, Fleming SM, Dolan RJ, Huys QJM. Different components of cognitive-behavioral therapy affect specific cognitive mechanisms. Sci Adv. 2024;10(13):eadk3222. 38536924 10.1126/sciadv.adk3222 PMC10971416

[17] Chisholm D, Sweeny K, Sheehan P, Rasmussen B, Smit F, Cuijpers P, Saxena S. Scaling-up treatment of depression and anxiety: a global return on investment analysis. Lancet Psychiatry. 2016;3(5):415–24. 27083119 10.1016/S2215-0366(16)30024-4

[18] Tong L, Miguel C, Panagiotopoulou OM, Karyotaki E, Cuijpers P. Psychotherapy for adult depression in low- and middle-income countries: an updated systematic review and meta-analysis. Psychol Med. 2023;53(16):7473–83. 37609800 10.1017/S0033291723002246 PMC10951412

[19] Li XM, Huang FF, Cuijpers P, Liu H, Karyotaki E, Li ZJ, et al. The efficacy of cognitive behavioral therapies for depression in China in comparison with the rest of the world: A systematic review and meta-analysis. J Consult Clin Psychol. 2024;92(2):105–17. 37902688 10.1037/ccp0000854

[20] Ying Y, Ji Y, Kong F, Wang M, Chen Q, Wang L, et al. Efficacy of an internet-based cognitive behavioral therapy for subthreshold depression among Chinese adults: a randomized controlled trial. Psychol Med. 2023;53(9):3932–42. 35388776 10.1017/S0033291722000599 PMC10317808

[21] Davidson B, Li DZ, Meng Y, Hamani C, Lipsman N. Psychiatric neuromodulation: the underappreciated importance of pre- and post-treatment care. Mol Psychiatry. 2021;26(2):366–9. 32724198 10.1038/s41380-020-0851-0

